# Eye involvement in a series of 94 young patients with congenital ichthyosis: Importance of early ophthalmological referral

**DOI:** 10.1016/j.jdin.2025.12.004

**Published:** 2025-12-23

**Authors:** Natalia Blanco-Calvo, Antonio Núñez-Reiz, Isabel Vals-Ferrán, Raman Malhotra, Angela Hernández-Martín

**Affiliations:** a Department of Ophthalmology, Hospital Infantil Universitario Niño Jesús, Madrid, Spain; b Hospital Universitario Clínico San Carlos, Madrid, Spain; c Ophthalmology and Oculoplastic Surgery Deparment, Corneoplastic Unit, Queen Victoria Hospital, East Grinstead, United Kingdom; d Department of Dermatology, Hospital Infantil Universitario Niño Jesús, Madrid, Spain

**Keywords:** age-related ocular risk, Congenital ichthyosis, dry eye disease, ectropion, epidermal differentiation disorder, genodermatosis, inferential statistics, keratinization disorder, ocular involvement

*To the Editor:* Congenital ichthyosis, now reclassified as epidermal differentiation disorders (EDDs), are hereditary conditions in which ocular involvement is frequently reported but has not been thoroughly characterized across subtypes.^1^^,^^2^ Although ectropion has traditionally been considered the most representative ocular manifestation,^3^^,^^4^ the broader spectrum of eye involvement and age-related progression in EDDs remains poorly defined. We sought to determine the prevalence and clinical relevance of ocular manifestations in children and young patients with EDDs and to explore potential causal relationships between disease features and ocular complications.

We conducted a prospective cross-sectional study in a multidisciplinary dermatology-ophthalmology clinic from January 2019 to June 2023. Approval from the Ethics Committee was obtained (R-0053-19). Ninety-four individuals (median age 9 years) with genetically confirmed EDDs were evaluated. EDD type, disease severity (Investigator global assessment 1-4),^5^ and complete ocular examinations were recorded. Demographic and clinical data are available at Supplementary Table I, available via Mendeley at https://data.mendeley.com/datasets/gpw7hmvhr2/1. Ocular findings are shown in Table I. Statistical analyses included univariate and multivariate logistic regression and a causal inference model to examine potential pathways linking disease characteristics to ocular outcomes (see Supplementary Material, available via Mendeley at https://data.mendeley.com/datasets/gpw7hmvhr2/1 for detailed description of methods).

**Table I:** Prevalence of ophthalmologic findings in our series (*N* = 94)

Anterior pole involvement	*n* (%)
Dry eye disease	68 (72)
Meibomian gland dysfunction	59 (63)
Exposure keratinopathy	29 (31)
Ectropion	26 (28)
Conjunctival injection	7 (7)
Corneal opacities	7 (7)
Total	71 (76)


Posterior pole involvement	Number (%)
Congenital glaucoma	1 (1)


Refractive disorders∗	Number (%)
Moderate to severe astigmatism	33 (37%)


Others	Number (%)
Infantile nistagmus	3 (3)

∗ Refractive disorders in 6 patients with corneal opacities could not be assessed.

Ocular involvement was highly prevalent, affecting 81% of patients, predominantly involving the eyelids and anterior segment (Supplementary Fig 1, available via Mendeley at https://data.mendeley.com/datasets/gpw7hmvhr2/1 and Supplementary Fig 2, available via Mendeley at https://data.mendeley.com/datasets/gpw7hmvhr2/1). Dry eye disease (DED) was the most frequent finding (72%), followed by meibomian gland dysfunction (MGD) (63%), exposure keratopathy (31%), and ectropion (28%). Corneal opacities were present in 7% of patients and were associated with reduced visual acuity. Refractive errors were common: moderate-to-severe astigmatism occurred in 37% and myopia in 18%. Only 1 patient presented posterior segment involvement (congenital glaucoma). Infantile nystagmus was observed in 3 patients (1 with trichothiodystrophy and 2 with *TGM1*-nEDD).

Ocular involvement varied by EDD subtype. Patients with *STS*-nonsyndromic EDD (nEDD) (formerly recessive X-linked ichthyosis) and *KRT1/KRT10* nEDD (formerly keratinopathic ichthyosis) exhibited significantly fewer ocular findings, whereas those with severe involvement and syndromic forms showed higher rates of DED, MGD, and ectropion. In univariate analysis, age was the strongest predictor of DED. In multivariate modelling, EDD type, but not severity, remained associated with ocular involvement, while age showed a positive trend toward increasing risk over time (Table II)

**Table II:** Comparison of dry eye disease, meibomian gland dysfunction and astigmatism according to IGA, sex and age

Variable (*n*)	DED *n* (%)	*P*-value	MGD *n* (%)	*P*-value	Moderate to severe astigmatism *n* (total)†	*P*-value
IGA						
IGA 1 + 2 (Mild) (53)	31	.01∗	26	.01∗	13 (52)	.01∗
IGA 3 (Moderate) (19)	16		13		9 (17)	
IGA 4 (Severe) (22)	21		20		11 (19)	
Sex						
Female (41)	33	.12	27	.586	23	.270
Male (53)	35		32		28	
Age						
<3 y (8)	3	.03∗	3	.122	5	.815
>3 y (86)	65		56		46	

Investigator Global Assessment: Mild = IGA 1-2; Moderate-Severe = IGA 3-4. p-value: Fisher exact test. *DED*, Dry eye disease; *MGD*, meibomian gland disfunction.

∗ Statistically significant.

† Astigmatism could not be assessed in 6 patients due to corneal opacities (1 patient with IGA 1-2, 2 patients with IGA 3, and 3 patients with IGA 4).

Causal inference analysis demonstrated a stepwise progression of eye disease: age-related MGD led to evaporative DED, which triggered conjunctival inflammation and keratopathy. Corneal opacities arose secondarily from chronic DED and exposure, and directly from ectropion (Supplementary Fig 3, available via Mendeley at https://data.mendeley.com/datasets/gpw7hmvhr2/1). Astigmatism was strongly associated with DED and ectropion, likely due to progression of corneal surface irregularity. Importantly, this model suggests that ocular morbidity in EDD is not solely a function of severity, but reflects cumulative effects of time, eyelid changes, and tear film instability.

These findings underscore the need for early and routine ophthalmological referral in EDD patients, even in the absence of overt ectropion, to detect and manage DED and MGD before irreversible corneal damage occurs. Patient education regarding ocular surface protection and regular monitoring of refraction are essential to prevent visual impairment. Follow-up studies with larger number of patients and longitudinal studies would be of great value.

## Conflicts of interest

Dr Hernández-Martín has the following COIs: Sanofi (advisory and speaking and lecture fees); Viatris (consulting or advisory and speaking and lecture fees); L'Oreal (consulting or advisory and speaking and lecture fees); LETI Pharma GmbH (speaking and lecture fees); Beiersdorf S A (speaking and lecture fees); with Galderma (consulting or advisory fees); Almirall (consulting or advisory fees), Alexion Pharma (speaker/Faculty education), and Celgene (co-investigator). Dr AHM declares that she has not known competing financial interests or personal relationships that could have appeared to influence the work reported in this paper.
